# Validation of a symphysis-fundal height chart developed for pregnancy complicated by diabetes and hyperglycemia: an observational study

**DOI:** 10.1186/s12978-016-0202-4

**Published:** 2016-08-03

**Authors:** Neusa Aparecida de Sousa Basso, Glilciane Morceli, Roberto Costa, Adriano Dias, Marilza Vieira Cunha Rudge, Iracema Mattos Paranhos Calderon

**Affiliations:** 1 Graduate Program in Gynecology, Obstetrics and Mastology, Botucatu Medical School, UNESP, Botucatu, Brazil; 2 Dr. Raul Bauab-Jahu Education Foundation, Jau, Brazil; 3 Integrated Faculties of Jaú, Jau, SP Brazil; 4 Department of Gynecology and Obstetrics, Botucatu Medical School, UNESP, Botucatu, SP Brazil; 5 Departamento Ginecologia e Obstetrícia, Faculdade de Medicina de Botucatu, UNESP, Distrito de Rubião Jr. s/n, Botucatu, Brazil 18618-970

**Keywords:** Fundal height, Risk pregnancy, Diagnostic validation, Diabetes, Hyperglycemia

## Abstract

**Background:**

The present study validates a symphysis-fundal height chart (SFH-chart) for pregnant women with type 2 diabetes mellitus (DM2), gestational diabetes mellitus (GDM) and mild gestational hyperglycemia (MGH) attending at the Diabetes and Pregnancy Reference Service of the Botucatu Medical School, UNESP, Brazil.

**Methods:**

A cross-sectional study was carried out to evaluate the performance of the specific FHC in predicting small (SGA) and large (LGA) for gestational age newborns (NB). We evaluated 206 pregnant women with DM2, GDM or MGH and their NB. The last symphysis-fundal height measure, taken at birth, was used to determine the sensitivity index (Sens), specificity index (Spe), positive prediction value (PPV), negative prediction value (NPV) and accuracy in predicting SGA and LGA. The gold standard was the Lubchenco birth weight/gestational age ratio evaluated at birth.

**Results:**

The mothers showed adequate glycemic control; 91.3 % of all pregnant women achieved HbA1c < 6,5 % in the third trimester. The SFH-chart tested achieved 100 % of Sens and NPV in predicting both SGA and LGA, with accuracy of 90.3 % (85.5; 93.6) and 91.8 % (87.2; 94.8), respectively, for predicting SGA and LGA newborns.

**Conclusions:**

The Basso SFH-chart showed high performance in predicting both SGA and LGA newborns of DM-2, GDM and MGH mothers, with better performance than the national reference SFH-chart. These findings support the internal validation of the Basso SFH-chart, which may be implemented in the prenatal care of the Diabetes and Pregnancy Reference Service-Botucatu Medical School/UNESP.

## Background

The reference symphysis-fundal height chart (SFH-chart) recommended by the Brazilian Health Ministry [1] was developed by the Latin American Center of Perinatology and Human Development (CLAP) and published in 1984 [2]. In developing countries, it is the primary if not the only tool for measuring fetal growth [3]. Other studies suggest the development of specific SFH-charts for each country, that is, based on the main population features [4–6]. As such, Brazilian studies have created new SFH-charts that are more suitable for their population [3, 7–15]. The most recent SFH-charts show significant contrasts in relation to the reference national SFH-chart [1, 2], which is more sensitive in identifying newborns that are small for gestational age (SGA-NB) [3, 13, 15].

Given the unsuitability of the national reference SFH-chart [1, 2] and the lack of specific SFH-charts for risk pregnancy, Basso [16] developed an SFH-chart for pregnancies complicated by diabetes and hyperglycemia. The Basso SFH-chart was based on 2470 symphysis-fundal height measures taken between 13 and 41 weeks of gestation, in 422 pregnant women with type 2 diabetes mellitus (DM2), gestational diabetes mellitus (GDM) [17] and mild gestational hyperglycemia (MGH) [18] under adequate glycemic control, with maternal glycemic mean < 120 mg/dL and/or HbA1c < 6,5 % [17, 18].

The Basso SFH-chart [16] differs significantly from the national reference SFH-chart [1, 2] and those developed by Oppermannn et al. [3] and Freire et al. [15] for Brazilians with low risk pregnancy. It was shown to be more appropriate than the others in predicting large for gestational age newborns (LGA-NB) because its percentiles exhibit higher values for most pregnancy weeks (P10 to P90). It is therefore more suitable for accompanying pregnancies complicated by DM2, GDM and MGH, which commonly result in larger babies. However, the diagnostic performance of the SFH-chart has yet to be tested on pregnant women with the aforementioned disorders [16]. In this sense, the present study aimed at validating the Basso SFH-chart [16] developed for pregnant women with DM2, GDM and MGH. To that end, the Basso SFH-chart was tested in a population that fits the described profile.

## Methods

### Design and subjects

**Fig. 1:**
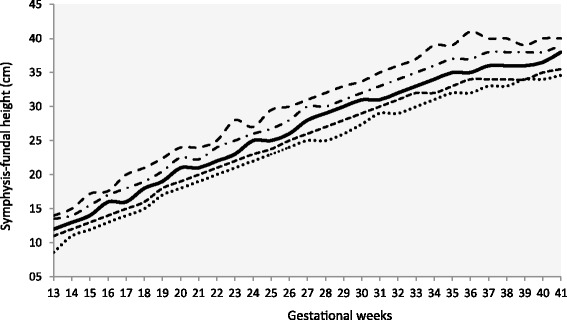
Basso SFH-chart [16] developed for pregnant women with type 2-DM, GDM or mild gestational hyperglycemia (MGH)

This observational study was carried out to validate the diagnostic performance of Basso SFH-chart [16] specific for pregnant women with DM2, GDM and MGH (Table 1 and Fig. 1). It was performed at the Diabetes and Pregnancy Reference Service of the Botucatu Medical School, UNESP, Sao Paulo, Brazil (SEDG-FMB/UNESP). The Human Research Ethics Committee of the Botucatu Medical School/UNESP approved the research project under protocol # 255/08.

**Table 1:** Expected value, lower and upper bounds of a 95 % confidence interval on Basso SFH-chart [16]* between 13 and 42 weeks of pregnancy complicated by diabetes and hyperglycemia

FH = 1.082 + 0.966*week			
FH (lower bound) = 0.629 + 0.95*week FH (upper bound) = 1.535 + 0.981*week			
		Confidence interval 95 %	
Week	Expected FH	Lower bound	Upper bound
13	13.64	12.98	14.29
14	14.61	13.93	15.27
15	15.57	14.88	16.25
16	16.54	15.83	17.23
17	17.50	16.78	18.21
18	18.47	17.73	19.19
19	19.44	18.68	20.17
20	20.40	19.63	21.16
21	21.37	20.58	22.14
22	22.33	21.53	23.12
23	23.30	22.48	24.10
24	24.27	23.43	25.08
25	25.23	24.38	26.06
26	26.20	25.33	27.04
27	27.16	26.28	28.02
28	28.13	27.23	29.00
29	29.10	28.18	29.98
30	30.06	29.13	30.97
31	31.03	30.08	31.95
32	31.99	31.03	32.93
33	32.96	31.98	33.91
34	33.93	32.93	34.89
35	34.89	33.88	35.87
36	35.86	34.83	36.85
37	36.82	35.78	37.83
38	37.79	36.73	38.81
39	38.76	37.68	39.79
40	39.72	38.63	40.78
41	40.69	39.58	41.76
42	41.65	40.53	42.74

All pregnant women attending at SEDG-FMB/UNESP in the period, and that met the inclusion criteria, were included in this study. As described in Basso study [16], the following inclusion criteria were adopted: (i) pregnant women with DM2, GDM or MGH; (ii) treated at SEDG-FMB/UNESP during the prenatal period (which involves at least five prenatal visit) and at birth; (iii) gestational age confirmed by early ultrasound (until 20 weeks); (iv) having a singleton pregnancy with live fetus; and (v) signing an informed consent form. A total of 206 pregnant women and their newborns were included in the study.

### Data collection

Data were obtained from the SEDG-FMB/UNESP database, which was updated daily with information acquired from every prenatal visit and hospitalization period, including the time of birth. Data were analyzed in Microsoft Excel 2003® spreadsheets.

Subjects were characterized according to maternal age (years), pre pregnancy body mass index (BMI) [19], number of gestations and gestational age at birth. The type of hyperglycemia condition was categorized according to Priscila White’s prognostic classification [20] and Rudge’s diagnostic criteria [18]. Glycated haemoglobin (HbA1c) was determined at late pregnancy to identify the quality of glucose control in the third trimester, considering levels < 6.5 % as adequate [17].

The SFH measures throughout pregnancy (13 to 41 weeks) were plotted on Basso SFH-chart [16]. The last measure, taken at birth, was used to calculate the values and confidence intervals (at 95 %) of the sensitivity index (Sens), specificity index (Spec), positive predictive value (PPV) and negative predictive value (NPV), and determine its accuracy in predicting small and large for gestational age newborns (SGA-NB and LGA-NB, respectively). The newborns were classified according to Lubchenco birth weight/gestational age ratio [21], which was used as gold standard to evaluate the Basso SFH-chart performance to predict SGA and LGA newborns.

### Subjects follow-up

According to SEDG-FMB/UNESP protocol [18], the diabetic pregnant women (type 2-DM) were immediately submitted to the glycemic control, with individual nutritional prescription and light to moderate-intensity exercises (walking 30 min five times a week), and received insulin from the first evaluation.

To diagnostic of GDM or MGH, were used the oral glucose tolerance test (75g-OGTT) and glycemic profile (GP), independently performed between 24 and 28 gestational weeks. The pregnant women with confirmed GDM or MGH were introduced to the same nutrition and exercise treatment protocol to achieve the glycemic control, and insulin was introduced when necessary [18].

The maternal glycemic control was evaluated by GP with fasting, pre- and post- prandial glycemic levels for 24 h in 2-week intervals until 32nd week, and weekly until delivery. A good glycemic control was achieved by glycemic mean < 120 mg/dL and/or HbA1c < 6.5 %. GDM or MGH pregnant women with adequate glycemic control and fetal growth waited for spontaneous labor until 39–40 weeks; those with no adequate glycemic control and/or fetal growth, and all type 2-DM have their delivery programmed about 37 weeks [18].

### Statistical analysis

The distribution of symphysis-fundal height measures on the Basso SFH-chart was performed using IBM SPSS Statistics 20.0 software. McNemar’s test evaluated the performance of Basso SFH-chart [16] and of the national reference SFH-chart [1, 2] in predicting SGA- and LGA-newborns.

## Results

The subjects profile (Table 2) shows that most were aged 25 years or older, with BMI corresponding to overweight and obesity, and at least one previous pregnancy. Of the 206 women analyzed, 104 (50.5 %) exhibited GDM and 29 (14.1 %) DM2, and according to Rudge diagnostic criteria [18], 73 (35.4 %) suffered from MGH. Mean maternal HbA1c level in the third trimester was 6.0 ± 1.06 %; 188 (91.3 %) pregnant women had adequate glycemic control, with HbA1c levels ≤ 6.5 %.

**Table 2:** Characterization of the pregnant women studied

	*N*	Frequency (%)
≥25 years of age	180	87.4
BMI ≥ 25 Kg/m^2^	152	73.8
≥1 previous delivery	146	70.9
Birth ≥ 37 weeks	189	91.7
P White classification^a^ [*n *= 133]		
A [diet controlled GDM]	83	62.4
A/B [diet + insulin controlled GDM]	21	15.8
B to C [DM2, no vascular diseases]	25	18.8
D to FRH [DM2, with vascular diseases]	4	3.0
Rudge groups^b^ [*n *= 206]		
IIA [abnormal GTT, normal GP]	15	7.3
IIB [abnormal GTT, abnormal GP]	118	57.3
IB [normal GTT, abnormal GP]	73	35.4
HbA1c < 6,5 % [3^rd^ trimester]	188	91.3

^a^Priscilla White’s prognostic classes [20] for diabetes in pregnancy: GDM (gestational diabetes) and DM2 (type 2 diabetes)

^b^Rudge’s diagnostic criteria for hyperglycemia in pregnancy [18] with the association glucose tolerance test (GTT) + glucose profile (GP)

IIA: GDM, with abnormal gestational GTT and normal gestational GP

IIB: GDM, with abnormal gestational TTG and GP; or DM2, with abnormal pre-pregnancy GTT

IB: mild gestational hyperglycemia (MGH), with normal gestational GTT and abnormal gestational GP

**Fig. 2:**
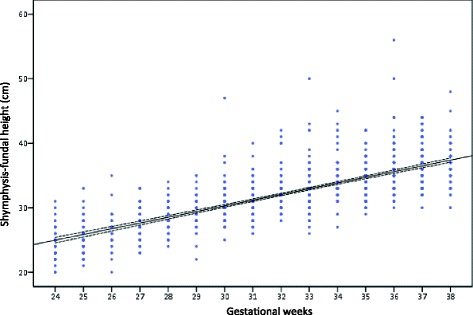
Distribution of 960 symphysis-fundal height measurements in this study plotted in Basso SFH-chart [16]

The women provided 980 symphysis-fundal height measures (mean = 4.76 measures each) between 24 and 38 weeks of pregnancy. The maximum number of measures per gestational age was 93 (for 34 weeks) and 104 (for 37 weeks). The measures were plotted on Basso SFH-chart [16] (Fig. 2).

Table 3 shows the distribution of the newborns according to Lubchenco birth weight/gestational age ratio [21]. The performance of Basso SFH-chart to predict SGA- and LGA-newborns showed 100 % of Sens and VPN for both SGA and LGA newborns. The accuracy of the Basso SFH-chart was 90.3 % (85.5; 93.6) for detecting SGA, and 91.8 % (87.2; 94.8) for identifying LGA. The Sens and NPV values on Basso SFH-chart [16] for predicting both SGA and LGA were higher than those on the national reference SFH-chart [1, 2] (Table 4).

**Table 3:** Newborn distribution into birth weight classes according Basso SFH-chart [16] and Lubchenco et al. [21]

	Lubchenco et al. [21]			
Basso SFH-chart [16]		SGA	N-SGA	Total
	SGA^a^	13	20	33
	Not SGA	0	173	173
	Total	13 (6.3 %)	193 (93.7 %)	206
	Lubchenco et al. [21]			
Basso SFH-chart [16]		LGA	N-LGA	Total
	LGA^b^	28	17	45
	No LGA	0	161	161
	Total	28 (13.6 %)	178 (86.4 %)	206

^a^SGA: small for gestational age newborn

^b^LGA: large for gestational age newborn

**Table 4:** Performance diagnostic to SGA- and LGA-newborns according to the Basso SFH-chart [16] and the national reference SFH-chart [1, 2]

	SFH-chart [16]		National reference SFH-chart [1, 2]	
	SGA^a^	LGA^b^	SGA	LGA
Sens	100,0 (77,2; 100,0)	100,0 (87,9; 100,0)	38.9 (20,3; 61,4)	66.7 (47,8; 81,4)
Espec	89,6 (84,5; 95,2)	90,4 (85,2; 93,9)	100,0 (96,3; 100,0)	100,0 (95,9; 100,0)
PPV	39,4 (24,7; 56,3)	62,2 (47,6; 74,9)	100 (64,6; 100,0)	100,0 (82,4; 100,0)
NPV	100,0 (97,8; 100,0)	100,0 (97,7; 100,0)	90,1 (83,1; 94,4)	91,0 (83,8; 95,2)
Accuracy	90,3 (85,5; 93,6)	91,8 (87,2; 94,8)	90,7 (84,1; 94,7)	92,4 (86,1; 95,9)
	SGA newborn [*X* ^2^ = 18.05 (*p* < 0,001)^c^]			
	LGA newborn [*X* ^2^ = 15.06 (*p* < 0,001)^c^]			

^a^SGA: small for gestational age newborn

^b^LGA: large for gestational age newborn

^c^McNemar’s Test

## Discussion

Our findings validate Basso SFH-chart [16], developed for DM2, GDM and MGH pregnant women with adequate glycemic control. The Basso SFH-chart [16] identified all the cases of intrauterine growth deviation, with respect to both growth restriction and excessive growth. The national reference SFH-chart [1, 2] showed a lower sensitivity index for predicting LGA-NB (66.7 %) and identified less than 40 % of SGA-NB (sensitivity = 38.9 %).

Compared to the results obtained with the national reference SFH-chart [1, 2], the most recent SFH-charts created for low-risk Brazilian pregnant women showed higher sensitivity in detecting SGA-NB [3, 13–15, 22]. The pioneering results of Belizan et al. [23] exhibited 86 % sensitivity, 90 % specificity and 10% false positive results in identifying growth-restricted fetuses, validating symphysis-fundal height measurements in the routine prenatal care. Thus, the findings of our study, with 100 % sensitivity, 89.6 % specificity, and no false positives in detecting SGA-NB, indicate the suitability of the Basso SFH-chart [16] for assessing growth restriction in pregnancies complicated with diabetes or hyperglycemia.

Regarding LGA-newborns, the Basso SFH-chart [16] identified all newborns with this condition, achieving 100 % of sensitivity; the sensitivity of the national reference SFH-chart [1, 2] was 66.7 %. In the Brazilian Study of Gestational Diabetes (EBDG), the distribution of symphysis-fundal height in the percentile 90 showed low sensitivity (0.8 a 6.0 %) to identify LGA-newborns [3]. The SFH-chart developed by Freire et al. [15] in Brazilian health pregnant women achieved sensitivity of 44.4 % and NPV of 89.4 % to predict LGA-newborns. Based on these national references, the indexes of 100 % sensitivity, 100 % NPV, 17 false positives (out of 178 cases), and no false negatives (28 cases) observed in our study, reinforce the good performance of Basso SFH-chart in identifying LGA-NB in pregnancies complicated with hyperglycemia.

All the best of our knowledge, SFH-chart specific for pregnancies complicated by diabetes or hyperglycemia had not yet been developed until Basso study [16]. This highlight the originality of our investigation, but difficult the data analysis. Basso SFH-chart [16] performed best in identifying both fetal growth restriction and excessive fetal growth in pregnancies complicated by diabetes and hyperglycemia. On the other hand, the most recent SFH-charts developed for the Brazilian population [3, 15] and the national reference SFH-chart [1, 2] did not exhibit adequate sensitivity indexes or NPV for this population. Considering that Sensitivity and PPV are indicators for a good diagnostic test [22], our results justify the use of Basso SFH-chart in the prenatal care at SEDG-FMB/UNESP.

However, some points must be reinforced. Similar to Freire et al. [15], the Basso SFH-chart [16] was based on data collected by a single observer, under controlled methodological procedures. These methodological features prevent the inter-observers bias. In the present study, although the same technical protocol, different professionals performed the symphysis-fundal height evaluation. According Oppermann et al. [3], this is a positive point to improve the efficiency and reproducibility of the Basso SFH-chart [16], and will likely contribute to its external validation.

Other point is that the subjects of Basso SFH-chart [16] have adequate glycemic control, and this characteristic was not a criteria inclusion in our study. However, our subjects and the population in the Basso study [16] was from a same health service, subjected to similar protocols for glycemic control, in general, resulting in adequate glycemic control. In our study, the HbA1c levels < 6.5 % in the end of gestation was achieved by 91.3 % (188/206) of all pregnant women. Besides, the statistic power calculation, considering glycemic control and gestational weeks, achieved 99.5 %. This findings support the internal validation of the Basso SFH-chart in our service.

Another question would be about the potential bias of pre- or pregnancy-BMI, and the necessity of an adjusting analysis by these variables. However, maternal BMI not seem to have been decisive in the original study [16]; the linear regression equation to predict SFH [SFH = 1.082 + 0.966*gestational week] showed that SFH varied only as a function of gestational age. Overweight or obesity is a common characteristic in diabetic pregnant women, constituting the physiopathologic base to insulin resistance, and this is a reality in our service. BMI ≥ 25 Kg/m^2^ was present in 73.8 % and 62,3 %, respectively, here and in Basso SFH-chart study [16]. BMI should be appreciated in external validation of the Basso SFH-chart in others services and subjects with different characteristics of BMI.

The Basso SFH-chart performance to predict LGA- and SGA-newborns was relative to late measures at birth, that is, at least 37 weeks in 91.7 % (189/206) of all cases. Our option was use the best (and real) gold standard, that is, the birth weight. Although not being the best gold standard for fetal growth [24], the measure of ultrasound abdominal circumference for each gestational week would be another option, but this cannot be included in our study. Either way, the distribution of our SFH measures, just superimposed on the Basso SFH-chart (Fig. 2), reinforces its validation.

Finally, the results of our study showed the high performance to predict the birth weight deviations of unique SFH-charts in pregnancies complicated by DM-2, GDM, and MGH. To clinical practice, the Basso SFH-chart [16] may be employed as a useful tool to C-section indications for macrosomia at the SEDG-FMB/Unesp. Another study using fetal ultrasound abdominal circumference as the gold standard should validate its use also to decisions on the maternal glycemic control during pregnancy. Likewise, other studies are needed to assess the reproducibility and external validation of Basso SFH-chart [16] for use in different diabetic pregnancy reference centers.

## Conclusion

The Basso SFH-chart [16] showed high performance in predicting both SGA and LGA newborns of DM-2, GDM and MGH mothers, with better performance than the national reference SFH-chart [1, 2]. These findings support the internal validation of the Basso SFH-chart [16], which may be implemented in the prenatal care of the Diabetes and Pregnancy Reference Service-Botucatu Medical School/UNESP.

## Abbreviations

SFH-chart, Symphysis fundal height chart; CLAP, Latin American Center of Perinatology and Human Development; GDM, gestational diabetes mellitus; MGH, mild gestational hyperglycemia; DM-2, type 2 diabetes mellitus; HbA1c, glycated haemoglobin; Sens, sensitivity index; Spec, specificity index; PPV, positive predictive value; NPV, negative predictive value, SGA-NB, small for gestational age newborn; LGA-NB, large for gestational age newborn

## References

[1] da Saúde M (2006). Pré-natal e puerpério – Atenção qualificada e humanizada. Secretaria de Atenção à Saúde. Departamento de Ações Programáticas Estratégicas.

[2] Fescina RH, Quevedo C, Martell M, Nieto F, Schwartz R (1984). Altura uterina como metodo para predecir el crescimiento fetal. Bol Oficina Sanit Panam.

[3] Oppermannn MLR, Duncan BB, Mengue SS, Ramos JGL, Serruya SJ, Schmidt MI (2006). Distribuição da altura uterina ao longo da gestação em uma coorte brasileira – comparação com a curva de referência do Centro Latino-Americano de Perinatologia. Rev Bras Ginecol Obstet.

[4] Buhmann L, Elder WG, Hendricks B, Rahn K (1998). A comparison of Caucasian and Southeast Asian Hmong uterine foundal height during pregnancy. Acta Obstet Gynecol Scand.

[5] Gardosi J, Francis A (1999). Controlled trial of fundal height measurement plotted on customised antenatal growth FHCs. Br J Obstet Gynaecol.

[6] Challis K, Osman NB, Nystrom L, Nordhal G, Bergstrom S (2002). Symphysis-fundal height growth FHC of an obstetric cohort of 817 Mozambican women with ultrasound-dated singleton pregnancies. Trop Med Int Health.

[7] Cunha SP, Ribeiro JU, Berezowski AT, Duarte G (1985). Evolução da altura uterina e circunferência abdominal em gestantes normais. Rev Paul Med.

[8] Silva JLP, Pereira B, Barini R, Reis C, Faúndes A (1986). Avaliação da curva de crescimento uterino na detecção de recém-nascidos pequenos para a idade gestacional. Ginecol Obstet Bras.

[9] Pedrosa de Freitas CB (1986). Evaluación de la altura uterina durante la gravidez. Ver Latino Am Perinatol.

[10] Barini R (1989). Avaliação da curva de crescimento da altura uterina como método para estimar o peso fetal [tese].

[11] Gouveia VL, Reis AFF, Amim Júnior J, Silva VL (1993). Valores normais da medida da altura do fundo de útero na gestação de 20 a 39 semanas. J Bras Ginecol.

[12] Pereira A, Gropen Júnior C, Lage EM, Cabral ACV (1997). Curva de crescimento da medida do útero-fita em gestações de risco habitual acompanhadas no Hospital das Clínicas–UFMG. J Bras Ginecol.

[13] Martinelli S, Bittar RE, Zugaib M (2001). Proposta de nova curva de altura uterina para gestaçöes entre a 20ª e a 42ª semana. Rev Bras Ginecol Obstet.

[14] Martinelli S, Bittar RE, Zugaib M (2004). Predição da restrição do crescimento fetal pela medida da altura uterina. Rev Bras Ginecol Obstet.

[15] Freire DMC, Paiva CSM, Coelho EAC, Cecatti JG (2006). Curva da altura uterina por idade gestacional em gestantes de baixo risco. Rev Bras Ginecol Obstet.

[16] Basso NAS. Evolução da curva de altura uterina em gestantes portadoras de diabete e hiperglicemia leve. 2013. [s.n.]. Tese (Doutorado) – Universidade Estadual Paulista-Unesp/FMB. [cited 20 February 2015] available at http://repositorio.unesp.br/bitstream/handle/11449/106618/basso_nas_dr_botfm.pdf?sequence=1.

[17] American Diabetes Association (ADA). Diagnosis and Classification of Diabetes Mellitus Diabetes Care. January 2012, 35(S1); S64–S71. 10.2337/dc12-s064 PMC3632174 22187472

[18] Rudge MVC, Calderon IMP, Ramos MD, Brasil MAM, Rugolo LMSS, Bossolan G (2005). Hiperglicemia materna diária diagnosticada pelo perfil glicêmico: um problema de saúde pública materno e perinatal. Rev Bras Ginecol Obstet.

[19] World Health Organization. BMI classification. 2004. Disponível em: http://apps.who.int/bmi/index.jsp?introPage=intro_3.html.

[20] White P (1978). Classification of obstetric diabetes. Am J Obstet Gynecol.

[21] Lubchenco LO, Hansman C, Dressler M, Boyd E (1963). Intrauterine growth as estimated from live born birth weight data at 24 to 42 weeks of gestation. Pediatrics.

[22] Bittar ER (2006). Distribution of uterine height along gestation in a Brazilian cohort–comparison with the reference curve of the Latin-American Center of Perinatology [EDITORIAL]. Rev Bras Ginecol Obstet.

[23] Belizán JM, Villar J, Nardin JC, Malamud J, De Vicurna LS (1978). Diagnosis of intrauterine growth retardation by a simple clinical method: measurement of uterine height. Am J Obstet Gynecol.

[24] Robert Peter J, Ho JJ, Valliapan J, Sivasangari S. Symphysial fundal height (SFH) measurement in pregnancy for detecting abnormal fetal growth. Cochrane Database of Systematic Reviews 2015, Issue 9. Art. No.: CD008136. DOI: 10.1002/14651858.CD008136.pub3. 10.1002/14651858.CD008136.pub3 PMC6465049 26346107

